# Bones Ejected from Her Body

**Published:** 1891-08

**Authors:** 


### Editorial, Etc.

BONES EJECTED FROM HER BODY; MISS SARAH HEAS, OF TENNESSSS, LOSES 600 PIECES; NEW ONES APPEAR.—Dr. B. F. Bell, of Parrettsville, Tenn., read before the East Tennessee Medical Society a paper on a wonderful case of exfoliation, or the ejection of bones from the human body. The victim of the peculiar disease is a lady named Sarah Heas, seventy-one years of age, residing in Caney Branch. About twenty-one years ago the doctor was first called to his patient and since that time he has given her ailment close attention. During all these years more than 600 b nes have been expelled from different parts of Miss Heas's body. This exfoliation, Dr. Bell said, has taken place without pain or inflammation, and soon after the ejection of the bones the fores would quickly heal up and nothing more would be noticed till another batch of bones would start out. The disease first began on the index finger of her left hand. Every bone in that hand disappeared and new ones took their places.

The next member of the body attacked was the arm; then the shoulder-blade, and then the lower jaw-bone. Some of the bones worked out through the throat and others penetrated the ear. The Doctor said the process worked so rapidly that he had sat near the patient many times and watched the bones as they came out without any assistance whatever except of nature itself. Some of them were six inches in length and one inch in diameter, while others were smaller. They were of every conceivable shape.

When the Doctor presented them to the fraternity for examination it appeared to be the contents of an unearthed coffin. But few of those present thought that they were anything else but bones from some lower animal. Doctor Bell says he will send the collection to an ostrological expert for examination. It has been six months since the first paper was read to the society on this remarkable case, and during this lapse of time the ejection has been going on at a rapid space.

In the latter part of his paper the Doctor said: "Three weeks ago I called to see the patient. While I was present he began to complain of severe pains in the regions of the coronoid process and extending to the ear. In less than five minutes from the beginning of pain a section of bone from the lower jaw, one inch long and one-quarter of an inch wide, passed through the intervening flesh and ligaments and presented itself thus entirely disengaged in the external left ear. Simultaneous with this was a similar section in the right ear, accompanied with a thin bone."

The Doctor concluded his paper with the following statement: The lady has resided in the same place for the past twenty-one years. She is visited by many persons who come to learn the real truth of the case, and they all go away declaring that it is the most wonderful phenomenon that has ever been heard of in this or any other land. The woman travels but little, and has never been on board a railroad train. She is well connected, having relatives in different parts of Tennessee.

